# Simulation of Membrane Fabrication via Solvent Evaporation and Nonsolvent-Induced Phase Separation

**DOI:** 10.1021/acsami.3c03126

**Published:** 2023-05-24

**Authors:** Niklas Blagojevic, Marcus Müller

**Affiliations:** Institute for Theoretical Physics, Georg-August University of Göttingen, 37077 Göttingen, Germany

**Keywords:** copolymer membranes, evaporation-induced self-assembly, nonsolvent-induced phase separation, simulation and modeling, micro- and macrophase separation

## Abstract

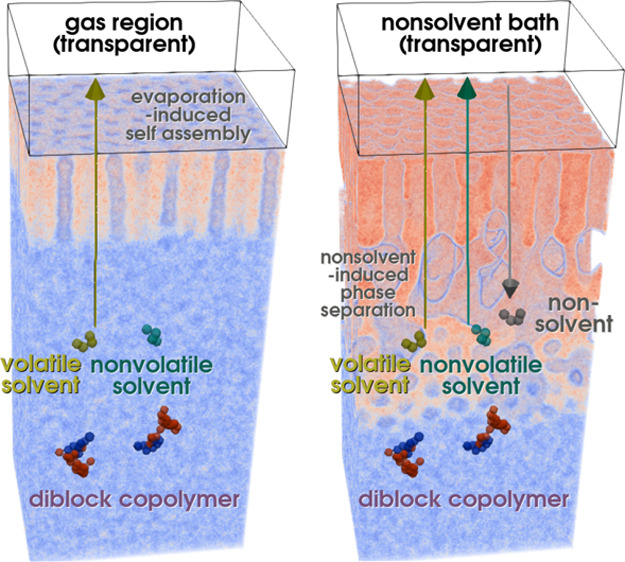

Block copolymer membranes offer a bottom-up approach to form isoporous membranes that are useful for ultrafiltration of functional macromolecules, colloids, and water purification. The fabrication of isoporous block copolymer membranes from a mixed film of an asymmetric block copolymer and two solvents involves two stages: First, the volatile solvent evaporates, creating a polymer skin, in which the block copolymer self-assembles into a top layer, comprised of perpendicularly oriented cylinders, via evaporation-induced self-assembly (EISA). This top layer imparts selectivity onto the membrane. Subsequently, the film is brought into contact with a nonsolvent, and the exchange between the remaining nonvolatile solvent and nonsolvent through the self-assembled top layer results in nonsolvent-induced phase separation (NIPS). Thereby, a macroporous support for the functional top layer that imparts mechanical stability onto the system without significantly affecting permeability is fabricated. We use a single, particle-based simulation technique to investigate the sequence of both processes, EISA and NIPS. The simulations identify a process window, which allows for the successful *in silico* fabrication of integral-asymmetric, isoporous diblock copolymer membranes, and provide direct insights into the spatiotemporal structure formation and arrest. The role of the different thermodynamic (e.g., solvent selectivity for the block copolymer components) and kinetic (e.g., plasticizing effect of the solvent) characteristics is discussed.

## 1 Introduction

Much effort in the field of industrial chemistry is devoted to separating the components of large quantities of chemical mixtures into pure or purer forms. The processes involved account for 10–15% of the world’s energy consumption.^1^ By virtue of its energy efficiency, membrane filtration has attracted significant attention as a promising alternative to thermal processes such as distillation.^2−4^

Specifically, porous membranes made of polymers and block copolymers have found multiple applications, such as ultrafiltration of functional macromolecules or colloids, and water purification.^5−14^ In particular, integral-asymmetric isoporous block copolymer membranes^15,16^ consist of a top layer of perpendicularly oriented pores with a narrow pore-size distribution that arises from the self-assembly of a cylinder-forming block copolymer. The selective top layer is supported by a macroporous substrate of the same material. This integral-asymmetric structure mitigates the selectivity permeability trade-off.^4^

The final membrane structure depends not only on the structure and thermodynamics of the constituents but also on the fabrication process;^17^ that is, the structure formation is kinetically trapped in the course of processing.^18^ The complex, nonequilibrium structure is formed by a sequence of two processes: (i) Initially, the top layer of perpendicularly oriented cylinders of the minority block is formed by evaporation-induced self-assembly (EISA) in a solution, comprised of an asymmetric block copolymer, a nonvolatile solvent, and a volatile solvent. (ii) Subsequently, the film is brought into contact with a coagulation bath, and nonsolvent-induced phase separation (NIPS) commences by the exchange of the nonsolvent and the remaining solvents in the film. In the course of this latter process, the functional top layer is preserved, and the macrophase separation between the nonsolvent and polymer results in the macroporous structure farther inside the film.

In accord with experiments,^16^ we employ two solvents: the volatile solvent, S, evaporates in the course of EISA, whereas the nonvolatile solvent, C, is exchanged during NIPS. The use of two solvents allows for (1) controlling the orientation of the microphase-separated morphology in the polymer skin (see section 3.1.1) and (2) tailoring the porous structure of the functional layer by swelling.^16^ Typical solvents include the following: tetrahydrofuran and dimethylformamide.^19^

To optimize permeability, selectivity, longevity, and cost, and to rationally design fabrication processes, direct insights into the spatiotemporal structure evolution in the entire course of the self-assembly and nonsolvent-induced phase separation (SNIPS) process—that is, the sequence of EISA and NIPS—are required.^12^ This remains a challenge for experiments,^12,20,21^ and molecular simulations can contribute to deriving a correlation between the molecular structure and thermodynamics, processing, and properties of the final nonequilibrium structure.

Note that the conditions that favor the formation of well-ordered perpendicularly oriented cylinders in the EISA process are not identical to those that benefit NIPS. For instance, if the volatile solvent is a strong plasticizer for the copolymer, the ordering kinetics of microphase separation may arrest in the course of EISA, resulting in a defect-riddled top layer. The kinetic arrest of the top layer, however, is necessary to stabilize the self-assembled top layer during the NIPS process.

Progress has been achieved for the individual processes, EISA or NIPS: Particle-based simulations and continuum models have investigated EISA of block copolymers, identifying factors that favor the formation and orientation of cylinders of the minority component perpendicular to the film surface: (1) fast evaporation^22−25^ (2) solvent selectivity for the matrix-forming, majority block^22−25^ as well as (3) preference of the film surface for the matrix-forming, majority block preventing layering of self-assembled structure.^24,25^ As the solvent evaporates, the polymer density at the film surface increases. Many aspects of the ordering can be rationalized by the time evolution of layers, in which (1) spherical micelles or (2) cylindrical domains can be formed.^25^

NIPS modeling approaches for *homopolymer* membranes have been reviewed in ref (12). Recently, Grzetic et al. studied the NIPS of copolymer films using dynamic self-consistent field theory (SCFT).^26^ In this study, the top layer that is formed by EISA in experiments is represented by an initial condition obtained by equilibrium SCFT. The authors studied the interplay between microphase separation between the two components of the block copolymer and macrophase separation between the nonsolvent and (co)polymer in the initial stage of NIPS. They observed that such films form the desired spongelike asymmetric porous substructure only if the solvent and nonsolvent have opposite block selectivities; that is, the solvent inside the film prefers the majority component, whereas the nonsolvent repels the majority component of the diblock copolymer stronger than the minority component.

The present work uses molecular simulation to investigate the entire SNIPS processes within a single, highly coarse-grained, particle-based model. This allows us to study the interplay between EISA and NIPS. Our paper is arranged as follows: In section 2 we introduce our soft, coarse-grained, particle-based model, the simulation technique, and a reference set of parameters that results in the formation of an integral-asymmetric isoporous block copolymer membrane. In section 3 we present first the details of the kinetics of the structure formation in the course of EISA and subsequent NIPS. Then we systematically vary thermodynamic characteristics as well as processing parameters to illustrate their role on SNIPS. The manuscript concludes with a brief summary and an outlook on future challenges.

## 2 Model and Simulation Technique

### 2.1 Structure and Thermodynamics of the Highly Coarse-Grained, Particle-Based Model

To access the long time and large length scales associated with the SNIPS process, we employ a highly coarse-grained, top-down model that accounts only for the universal characteristics of polymer–solvent systems. The system is comprised of 4 molecular species: (1) an asymmetric *AB* diblock copolymer, whose *A* fraction *f*_*A*_ = 0.3125 results in the formation of cylindrical *A* domains, (2) a volatile solvent, *S*, (3) a nonvolatile solvent, *C*, and (4) a nonsolvent, *N*. In accord with previous work,^25^ the liquid–vapor coexistence of the compressible polymer–solvent system is replaced by a phase coexistence between polymer–solvent and gas, *G*.

**Figure 1:**
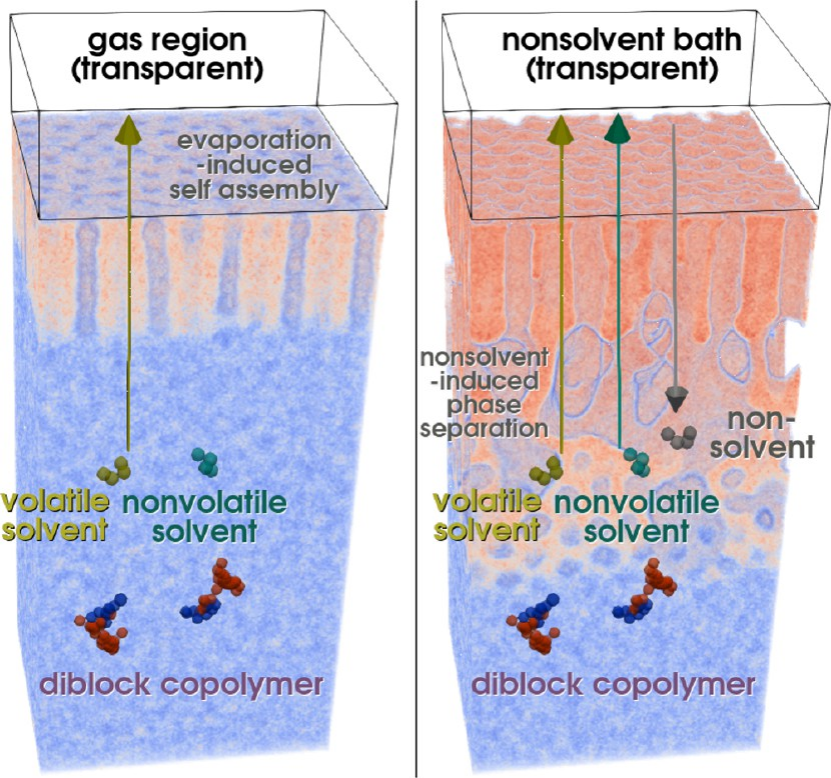
Sketch of the simulation setup for the SNIPS process: For the simulation of the first step of SNIPS, EISA (left), we convert volatile solvent molecules that diffuse beyond the top of the film into gas molecules. This leads to the formation of a self-assembled top layer with perpendicular cylinders, as the solvent density at the top of the film decreases and the polymer density increases, in turn. In the second step, NIPS (right), we bring the top of the film into contact with a nonsolvent bath. The nonsolvent molecules exchange with both remaining solvents in the film. This leads to the creation of a macroporous structure beneath the self-assembled top layer by macrophase separation between the nonsolvent and polymer.

We use a system of size *V* = *L*_*x*_ × *L*_*y*_ × *L*_*z*_ = 13.8 × 16 × 50*R*_e_^3^ for most of our simulations. *R*_e_ denotes the end-to-end distance of the block copolymer. For the calculation of the characteristic lateral domain size of the macropores (section 3.1.3), however, we quadruple the lateral extent of the *xy* plane. The evaporation proceeds into the negative *z*-direction. A sketch of the molecules and system setup is shown in Figure 1. Periodic boundary conditions are applied in the two lateral directions, *x* and *y*, whereas walls confine the system at *z* = 0 and *L*_*z*_.

A segment of our particle-based model represents multiple repeat units along the molecular backbone. We distinguish between strong bonded interactions that define the molecular shape and weak nonbonded interactions that dictate the thermodynamics of mixing.^27−29^ The bonded interactions take the form
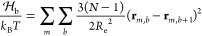
1The first sum runs over all molecules, *m* = *AB*, *S*, *C*, *N*, and *G*, whereas the second sum enumerates all bonds between neighboring segments, *b* and *b* + 1. The discretization *N* = 32 denotes the number of segments of the *AB* diblock copolymer, and *R*_e_ characterizes its root mean square end-to-end distance in the absence of nonbonded interactions. All other molecular species are comprised of 4 bonded segments. We represent the smaller molecules as oligomers with 4 segments, instead of single-bead units because, first, the single-chain-in-mean-field (SCMF) algorithm^27,30^ which we employ for our simulations (see section 2.2) exploits the scale separation between the strong bonded and weak nonbonded forces. The increase of the discretization, *N*, of the molecular contour allows us to use weaker nonbonded interactions per bead and, in turn, increases the strength of the bonded interactions if the molecular size remains unaltered. The second reason consists of the evaluation of the nonbonded interactions via a collocation grid, in conjunction with the simple assignment of a bead to the density of the nearest grid cell, *vide infra*. Since only beads within a grid cell interact, nonbonded interactions do not give rise to square-gradient terms in the corresponding continuum theory; that is, an immiscible mixture of two monomer species would not coarsen into macroscopic domains. Representing the solvent by oligomers, instead, couples neighboring grid cells and results in macrophase separation.

The nonbonded interactions are expressed as a function of the local normalized densities, ϕ̂_*i*_(*c*) that are defined on a cubic collocation grid of linear spacing Δ*L* = *R*_e_/10.
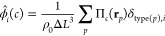
2where the sum runs over all segments that can adopt the types *i* = *A*, *B*, *S*, *C*, *N*, and *G. Π*_*c*_(**r**_*p*_) = 1 if the segment *p* at position **r**_*p*_ is inside the grid cell *c*, and 0 otherwise. The hat indicates that the densities are calculated from the explicit molecular configurations. ρ_0_ denotes the number density of segments. To quantify the density, we employ the invariant degree of polymerization of the copolymer melt, , and use the experimentally large value .

The nonbonded energy takes the form
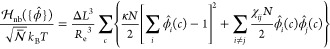
3where the first term restrains fluctuations of the local segment density ∑_*i*_ϕ̂_*i*_(*c*) around the reference value 1, and *κN* = 150 is related to the inverse, isothermal compressibility of the polymer–solvent–gas system. χ_*ij*_ denote the Flory–Huggins parameter between the different segment species. The explicit description of the gas allows us to control the surface tension of the film surface and its preference for the species via the Flory–Huggins parameter, χ_*Gi*_, rather than by balancing short-range repulsions and longer ranged attractions of the species *A*, *B*, *S*, *C*, and *N*.

### 2.2 Simulation Technique: Local Density-Dependent Mobility and Evaporation/Solvent Exchange

The molecular configurations are updated by Monte Carlo (MC) simulations. In order to exploit the difference between the strong bonded and weak nonbonded interactions, we utilize the SCMF algorithm^27,30^ that temporarily replaces the slowly changing but computationally expensive nonbonded interactions by quasi-instantaneous external fields.

The segment coordinates are updated by a smart Monte Carlo algorithm that uses the strong bonded interactions to propose a local random segment displacement.^31^ This results in Rouse-like dynamics.^32^ An *AB* diblock polymer in a disordered, spatially homogeneous melt, χ_*AB*_*N* = 0, requires τ_R_ = 6880 Monte Carlo sweeps to diffuse its own end-to-end distance, that is, τ_R_ = *R*_e_^2^/*D* = 3π^2^ τ_Rouse_ with *D* and τ_Rouse_ being the single-chain self-diffusion coefficient and the Rouse time, respectively.

As the solvent evaporates or the nonsolvent and polymer macrophase separate, the polymer density increases, and the polymer dynamics arrest in a glassy state. The need to capture this glassy arrest can be illustrated, for example, by considering the film after the EISA process. At this stage, the evaporation of the volatile solvent, *S*, resulted in the formation of a polymer-rich skin at the film surface in which the block copolymer self-assembled into a well-ordered layer of *A*-core cylinders. If the film equilibrated at this stage, the remaining nonvolatile solvent, *C*, would dissolve the polymer skin, thereby destroying the self-assembled top layer. Thus, the glassy arrest of the thin top layer is necessary to increase the temporal process window for switching from EISA to NIPS. The glassy arrest has also been implicated in the degree of pore-size asymmetry in the NIPS process for homopolymers.^33^

In our soft, coarse-grained model, there are no significant liquid-like packing effects of segments, and we account for the plasticizing effect of the solvents by a mobility modifier, 0 ≤ *m*_*i*_({ϕ̂(*c*)}) ≤ 1.^25,27^ It allows us to control the local dynamics of a segment of type *i* as a function of the local environment, without altering the thermodynamic equilibrium properties. To this end, we modify the original acceptance probability, *p*_acc_^0^(**r** → **r**′), of a proposed segment displacement from position **r** in cell *c* to **r**′ in *c*′ to
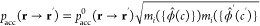
4where {ϕ̂(*c*)} and {ϕ̂′(*c*′)} denote the densities before and after the MC trial move, respectively. This modified acceptance probability does not alter the detailed balance.

We simulate the system in the semi-grand-canonical ensemble; that is, the total number of segments is constant, but solvent molecules, *S* or *C*, are converted into gas, *G*, in the case of EISA^23−25,27,34^ or nonsolvent, *N*, in the case of NIPS at a distance above the film surface. This distance is set to *d*_EISA_ = 2*R*_e_ for solvent evaporation and *d*_NIPS_ = 1.3*R*_e_ for nonsolvent–solvent exchange, respectively. In the course of EISA, the film surface moves downward as the volatile solvent evaporates, but the gas does not enter the film, and the conversion zone dynamically follows the film surface. In agreement with prior studies,^25^*d*_EISA_, results in an experimentally relevant Péclet number (see section 3.1.1).

The SCMF algorithm including the mobility modifier and the molecular conversion of solvents, nonsolvent, and gas are efficiently implemented in the graphics processing unit (GPU)-accelerated program soft coarse-grained Monte Carlo acceleration (SOMA) that allows us to study large length and long time scales.^29,35^

### 2.3 A Common Parameter Set for EISA and NIPS

Even for this highly coarse-grained, top-down model there is a high-dimensional parameter space of structural, thermodynamic, and processing parameters. In the following we keep the structural parameters, such as the fraction, *f*_A_, constant but vary the thermodynamic parameters, χ_*ij*_*N*, the polymer concentration in the film, and the duration of the EISA process. In this section, we provide a set of common parameters for EISA and NIPS that result in the *in silico* formation of integral-asymmetric, isoporous membranes via SNIPS. This point in the high-dimensional parameter space is robust, and variations of parameters away from this reference system are discussed in section 3.2. The set of reference parameters, however, is neither adapted to specific experiments nor aims at optimizing specific properties of the final membrane structure.

The initial densities in the polymer film at the start of the EISA process are ϕ_*P*_0__ = ϕ_*A*_0__ + ϕ_*B*_0__ = 0.387, ϕ_*S*_0__ = 0.293, and ϕ_*C*_0__ = 0.320. The homogeneous initial film does not contain gas or nonsolvent. The symmetric matrix of incompatibilities of the reference system takes the form
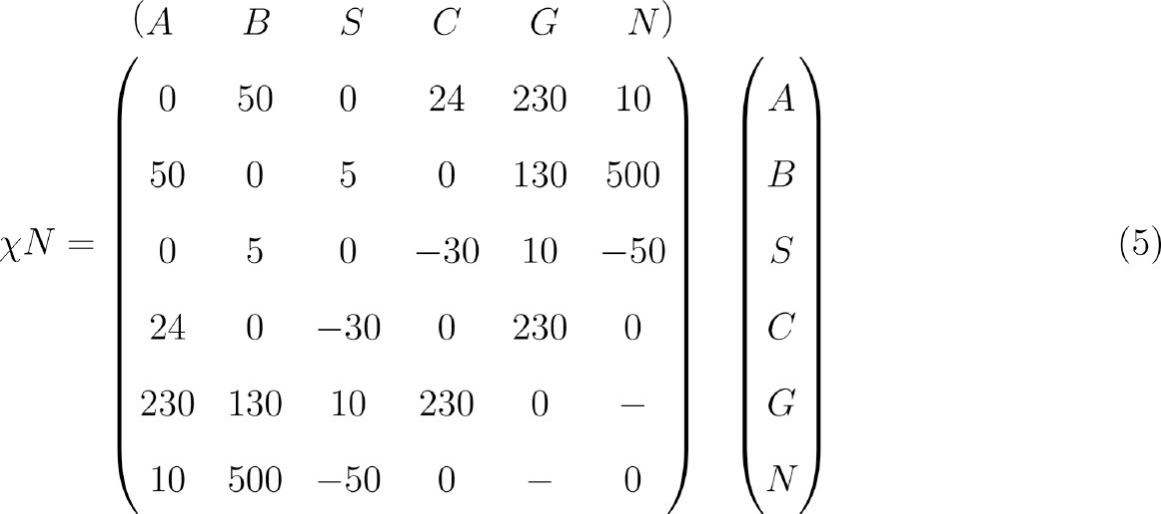
5Note that χ_*AB*_*N* is the appropriate invariant quantity that characterizes the incompatibility between the blocks of the diblock copolymer (independently of the molecular weight of the copolymer). The incompatibility between the blocks of the copolymer is χ_*AB*_*N* = 50, well above the incompatibility at the order–disorder transition (ODT) of the pure diblock copolymer melt.^36^

The solvents, *S* and *C*, are miscible with the polymer and the volatile solvent, *S*, is slightly selective for the cylinder-forming block, *B*, whereas the nonvolatile solvent is selective for the matrix-forming block, *A*. Note that the matrix entries correspond to χ_*ij*_*N* with *N* = 32 being the contour discretization of the block copolymer in order to be consistent with eq 3; the miscibility of solvents and solvent and polymer, however, involves the binary interaction strength, χ_*ij*_, but is independent from the polymer chain contour discretization, *N*.

The nonsolvent, *N*, is highly incompatible with *B*, whereas there is weak repulsion between the nonsolvent and *A*. The volatile solvent, *S*, strongly prefers the nonsolvent. This leads to a strong flux of the remaining volatile solvent after EISA into the nonsolvent bath, where it becomes converted to nonsolvent molecules. This rapid decrease of ϕ_*S*_ contributes to the stabilization of the microphase-separated polymer skin at the beginning of the NIPS process by glassy arrest, *vide infra*.

The nonvolatile solvent, *C*, is compatible with the nonsolvent. The interaction between gas and nonsolvent needs not be specified because these two species are not simultaneously present in the system.

Qualitatively, the chosen solvent selectivities of the reference system are in accord with a recent SCFT study of NIPS by Grzetic et al., who found that the desired spongelike macrophase-separated structure forms only if the nonsolvent, *N*, and the (remaining, nonvolatile) solvent, *C*, have opposite block selectivities and choose the solvent to be selective for the majority block, *B*. It remains, however, of interest to study alternate solvent selectivities in the future.

For the dependence of the segment mobility on the local densities, we use the mobility modifier
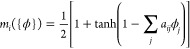
6with mobility-coefficient matrix
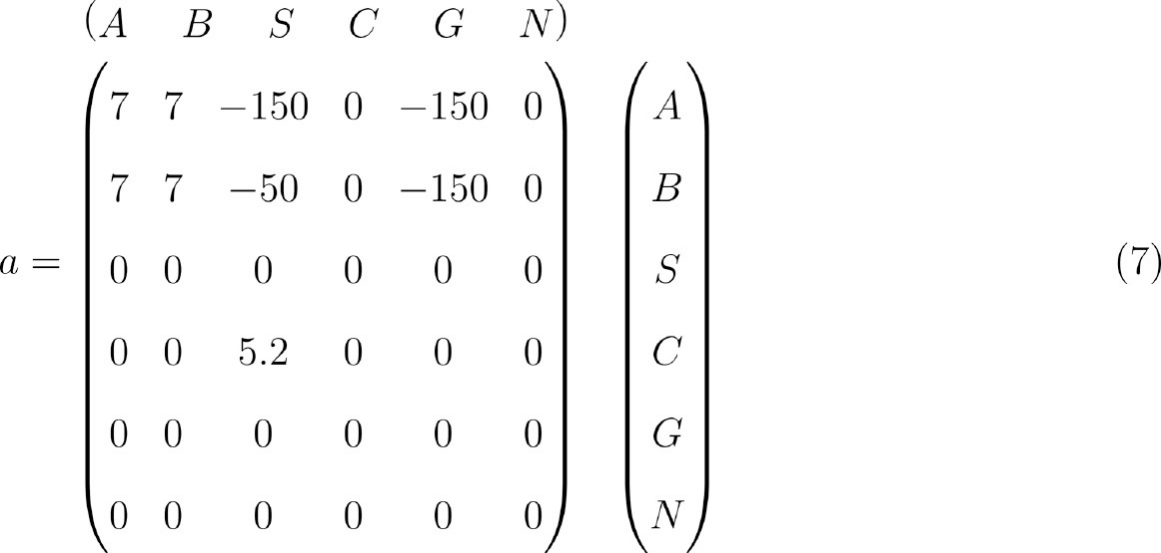
7A functional form similar to eq 6 has also been employed in a recent continuum model.^26^

**Figure 2:**
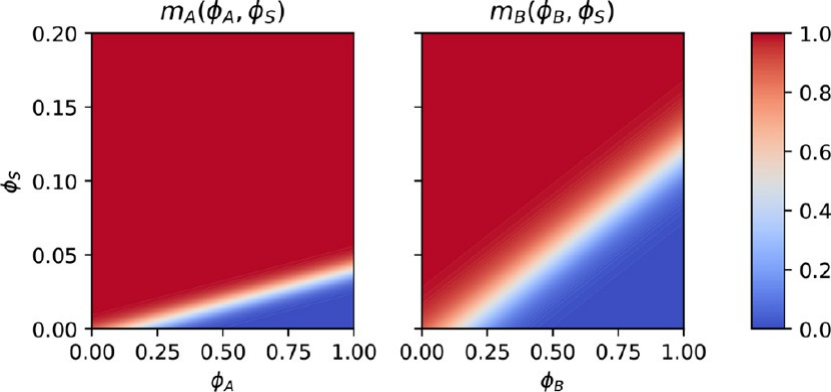
Dependence of the mobility modifier, eq 6, for *A* and *B* segments as a function of the local densities of the same block and the volatile solvent, *S*. The density of the other segment species is set to 0.

The mobility of polymer segments, *A* and *B*, decreases if the local polymer density increases (glassy arrest), *a*_*AA*_ = *a*_*BB*_ = *a*_*AB*_ = *a*_*BA*_ = 7 > 0, but increases by the presence of volatile solvent, *S*, or gas, *G*, as represented by negative mobility coefficients. This dependence is illustrated in Figure 2. The solvent *S* is a better plasticizer for the minority component *A* than for *B*. We do not consider the dependence of the polymer mobility on the nonsolvent density because polymer and nonsolvent are immiscible. The mobility of volatile solvent, gas, and nonsolvent are independent of the local density, whereas the mobility of the nonvolatile solvent slightly decreases in the presence of the solvent *S*. The latter effect turns out to be beneficial for the formation of perpendicular cylinders in the course of EISA, *vide infra*.

## 3 Results and Discussion

### 3.1 SNIPS for the Reference System

#### 3.1.1 EISA

The addition of a second, nonvolatile solvent, *C*, increases the parameter space of EISA. Thus, a delicate choice of system parameters and processing protocol is necessary to obtain the desired, perpendicular cylinder morphology at the top of the film.

**Figure 3:**
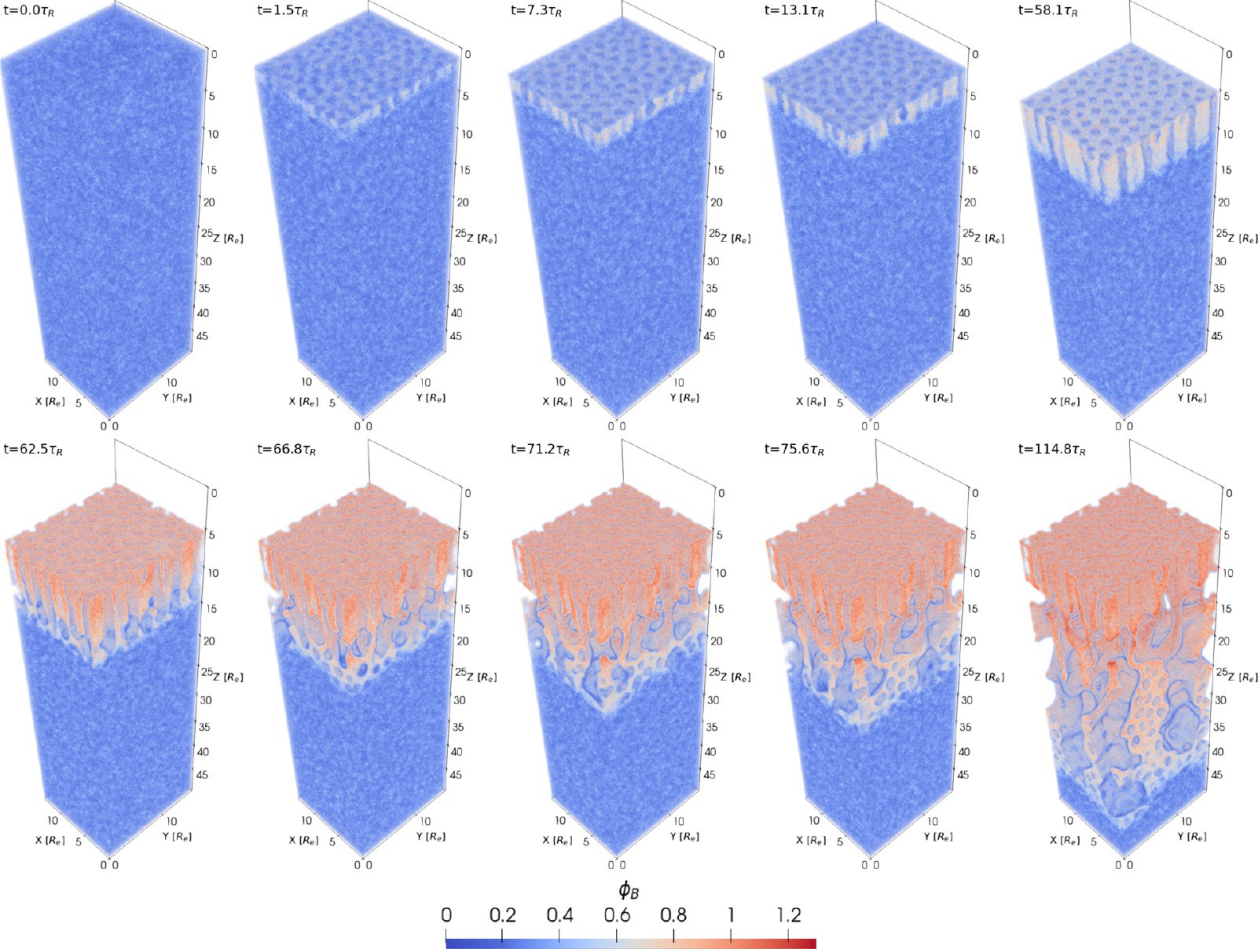
Snapshots of the majority-block density, ϕ_*B*_, during SNIPS at different times. The EISA process continues until *t* = 58.1τ_*R*_. Thereafter, the NIPS process commences.

**Figure 4:**
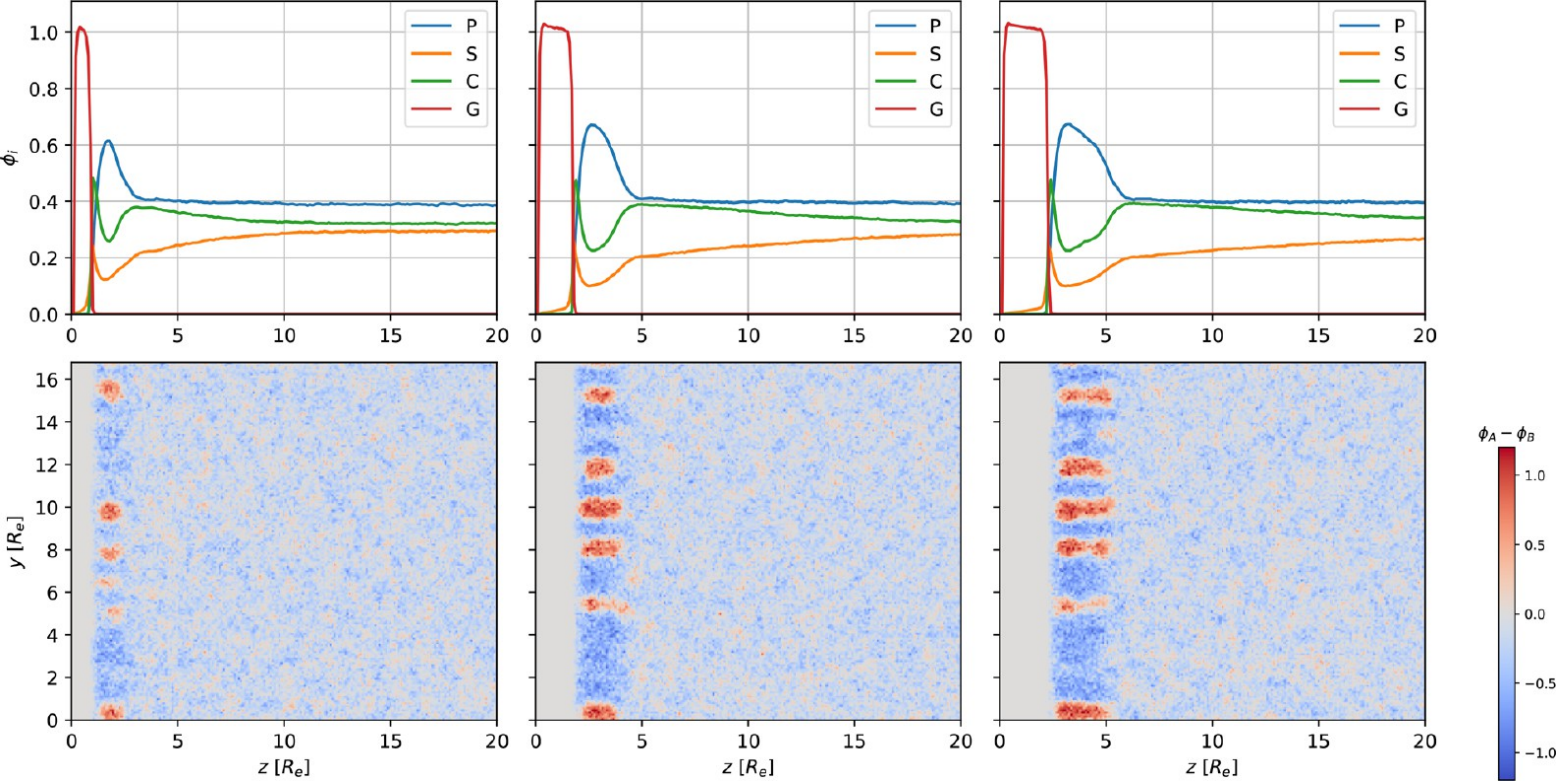
Top: 1D density profiles of polymer, *P* = *A* + *B*, volatile solvent, *S*, nonvolatile solvent, *C*, and gas *G*, at the top of the system as a function of the perpendicular position, *z*. Bottom: two-dimensional (2D) cross section of the density difference, ϕ_*A*_ – ϕ_*B*_, in the corresponding region. The snapshots show the systems at times *t*/τ_*R*_ = [1.5, 7.3, 13.1] from left to right. Three-dimensional (3D) snapshots of the majority-block density at the corresponding times are shown in Figure 3.

Figure 3 and Figure 4 present the formation of perpendicular cylinders of the minority component, *A*, in the course of EISA. As the volatile solvent, *S*, evaporates, the film surface moves downward (to the right in Figure 4). The depletion of *S* (and also of *C* because χ_*SC*_*N* = −30) at the film surface and the movement of the surface cause the polymer to enrich at the retracting surface. A polymer-rich layer (skin) forms and subsequently extends away from the film surface.

Due to the increase of the polymer density, the ODT is crossed, and the polymer microphase separates. Initially, *t* = 1.5τ_*R*_, spherical micelles self-assemble at the top surface as the thickness of the polymer skin reaches about *R*_e_. In the course of evaporation, *t* = 7.3τ_*R*_ and 13.1τ_*R*_, the polymer skin grows thicker, and the micelles elongate perpendicularly to the film surface into *A*-rich cylinders that laterally arrange onto a hexagonal lattice. (If the ODT is reached too early, e.g., due to a small *d*_EISA_ or χ_*SG*_*N*, it may happen that a layer of micelles forms that laterally fuse into parallel cylinders.)

The simulation of the reference system results in a nearly defect-free top layer of perpendicular cylinders. The length of these cylinders can be controlled by the duration of the EISA process.

There are several aspects in which EISA with a volatile and a nonvolatile solvent differs from the single-solvent process: The evaporation of the volatile solvent, *S*, generates a gradient inside the film. Unlike the single-solvent case, ϕ_*C*_ = 0,^25^ this gradient is not compensated by a concomitant opposite gradient of the polymer density (due to near-incompressibility) but, instead, by a gradient of the nonvolatile solvent, *C*. Since the nonvolatile solvent diffuses faster than the copolymer, it can compensate for the loss of solvent farther inside the film.

This results in a rather narrow, well-defined front between the self-assembled copolymer skin with a density of ϕ_*P*_ = ϕ_*A*_ + ϕ_*B*_ ≳ 0.6 and the disordered interior of the film with spatially constant ϕ_*P*_ = 0.387. Thus, the layer beneath the self-assembled top layer of perpendicular cylinders, where the polymer density suffices to form micelles but not to transform micelles into cylinders, is smaller than *R*_e_, favoring the perpendicular growth of cylinders into the disordered interior of the film, according to the layer-evolution model.^25^

The polymer-skin formation is enhanced by the attraction, χ_*SC*_*N* = −30, between *S* and *C*. Then the reduced density, ϕ_*S*_, of the volatile solvent at the film surface gives rise to an additional reduction of ϕ_*C*_ and, by virtue of the near-incompressibility, a concomitant increase of polymer at the film surface (skin formation). Moreover, the depletion of nonvolatile solvent, *C*, leads to a slight enrichment behind the microphase-separated polymer skin and to a corresponding decrease of polymer density due to near-incompressibility in this region. (For χ_*SC*_*N* = 0 (data not shown), the density, ϕ_*P*_, inside the polymer skin remains smaller, and the interface between the polymer skin and the interior of the film is more gradual. Therefore, multiple layers of spherical micelles form.) Moreover, as the nonvolatile solvent, *C*, is pushed away from the film surface, its density increases beneath the self-assembled top layer. The diffusion farther downward, however, is slowed down because the mobility of *C* decreases with increasing ϕ_*S*_. Thus, there is a density gradient of *C* with a sign opposite to that of the volatile solvent *S*. In turn, the polymer density after the skin layer remains almost constant.

Thus, the parameters, χ_*SC*_*N* and *a*_*CS*_, allow for controlling the polymer density inside the skin and the formation of a rather narrow front between the self-assembled skin and the disordered interior of the film.

**Figure 5:**
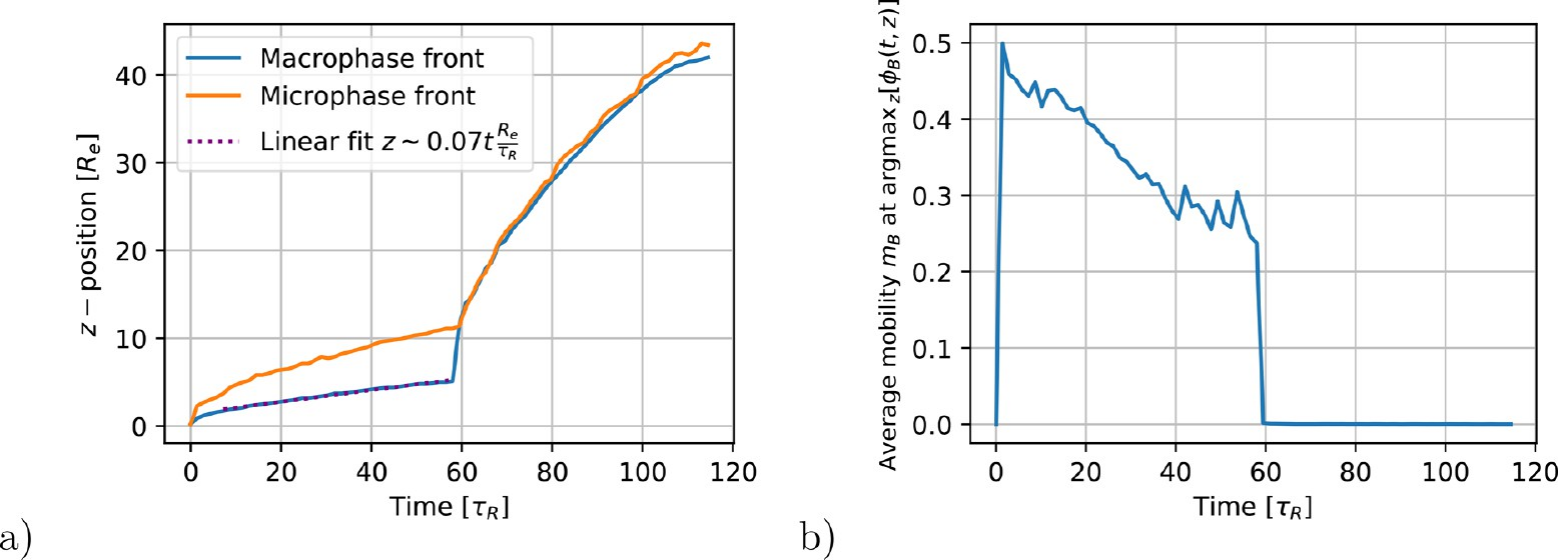
(a) Micro- and macrophase-separation-front positions as a function of time. The switch from EISA to NIPS occurs at t* = 58.1τ_*R*_. For *t* < t*, macrophase separation refers to the coexistence between polymer film and gas (vapor); i.e., the corresponding front is the film surface. In the course of NIPS, *t* >t*, macrophase separation refers to the coexistence between nonsolvent and polymer-rich domains. During NIPS the film surface only very slightly retracts. (b) Average mobility of the matrix block, *B*, in a cross-sectional slice at the position of the maximum of the 1D density profile argmax_*z*_[ϕ_*B*_(*t*, *z*) ]. For the calculation of the average mobility we consider only regions in the cross section where ϕ_*B*_ > 0.4.

The time evolution of the perpendicular position of the film surface (or front of the macrophase separation between the polymer film and gas) and the interface between the polymer skin and the disordered interior of the film is presented in Figure 5a. We use a linear fit in the interval 7.3τ_*R*_ ≤ *t* < 58.1τ_*R*_ to estimate the velocity, *v*, with which the film surface retracts. The corresponding dimensionless Péclet-Number, *Pe* = *vτ*_*R*_ /*R*_e_ ≈ 0.07, indicates that the evaporation is rather fast.

In Figure 5b, we present the average mobility of the matrix block in a cross-sectional slice at the position of the maximum of its 1D density as a function of time. During EISA, we observe an approximately uniform decrease of polymer mobility, which results from a steady increase of polymer density at the top of the film and a concomitant decrease of volatile-solvent density at that location.

The additional, nonvolatile solvent, *C*, can be used to change the ODT and the mechanisms of cylinder growth. The volatile solvent, *S*, slightly prefers the minority block, *A*, whereas the nonvolatile solvent, *C*, has a preference for the majority component *B*. This differs from EISA with a single solvent,^22−25^ where perpendicular cylinders are formed if the volatile solvent prefers the matrix-forming block, *B*.

Note that the concentration of the nonvolatile solvent, *C*, always remains larger than that of the volatile component, *S*. Moreover, inside the polymer skin, the density profiles of ϕ_*S*_ and ϕ_*C*_ exhibit a similar spatial dependency. Thus, in the region where the copolymer self-assembles, we can roughly approximate the solvent mixture by a single solvent that favors the majority block, *B*. This allows for a somewhat larger process window, and we also observe perpendicular cylinders when the volatile solvent, *S*, is neutral, i.e., 0 ≤ χ_*AS*_*N* = χ_*BS*_*N* ≤ 5.

Further inside the film, however, the spatial dependencies of ϕ_*S*_ and ϕ_*C*_ are opposite, i.e., ϕ_*C*_ reaches the value deep inside the film from above, whereas ϕ_*S*_ approaches its asymptotic value for large *z* from below.

#### 3.1.2 NIPS

**Figure 6:**
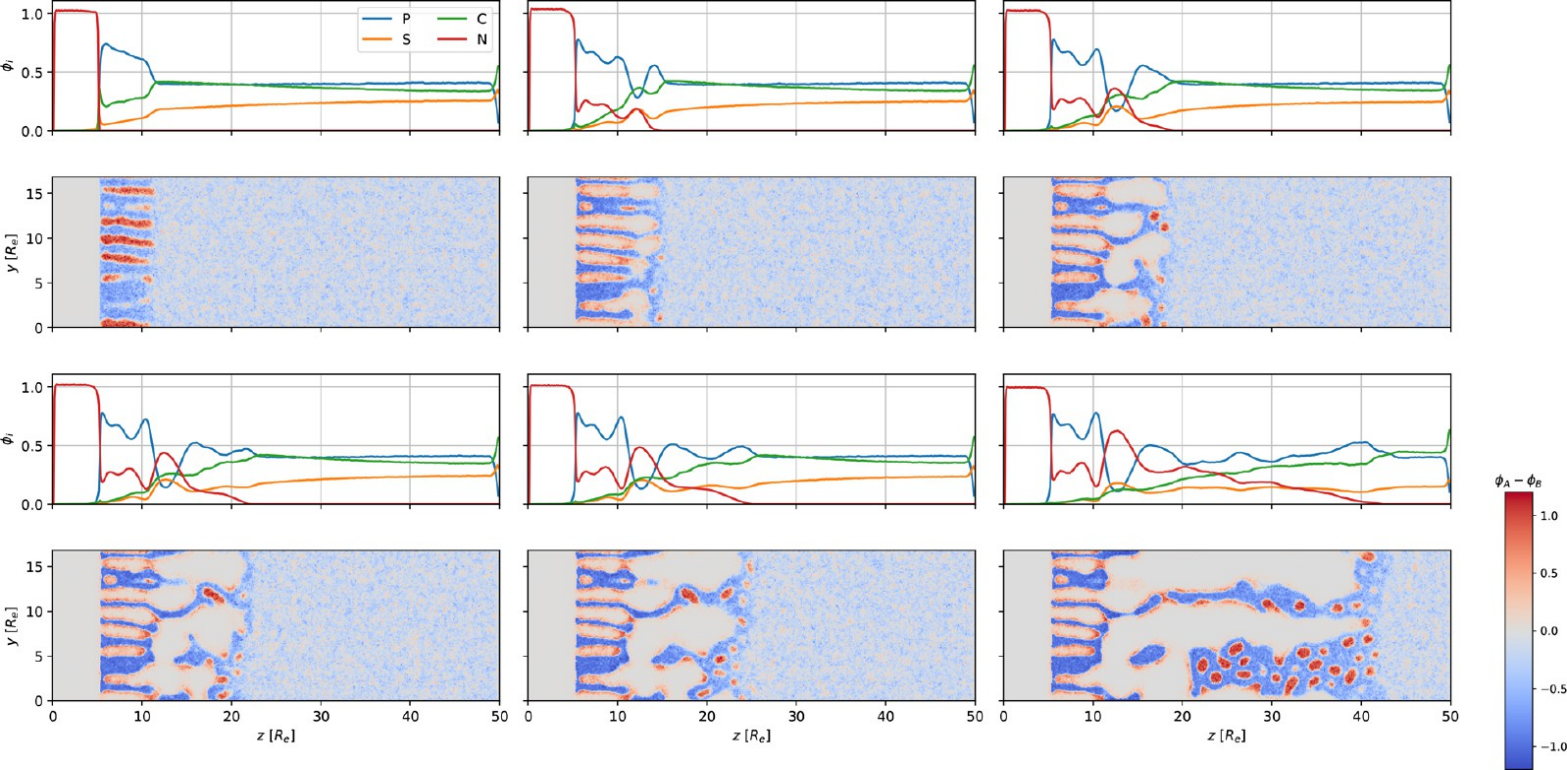
First and third row: 1D density profiles of polymer, *P* = *A* + *B*, solvents *S* and *C*, and nonsolvent, *N*, as a function of the perpendicular position, *z*. Second and fourth row: 2D cross section of the difference, ϕ_*A*_ – ϕ_*B*_, between minority-block and majority-block density corresponding to the 1D graphs above. The images from top left to bottom right show the systems at times *t*/τ_*R*_ = 58.1, 62.5, 66.9, 71.2, 75.6, and 114.8. The NIPS process commences at *t* = 58.1τ_*R*_. 3D snapshots of the majority-block density at the corresponding times are shown in Figure 3.

**Figure 7:**
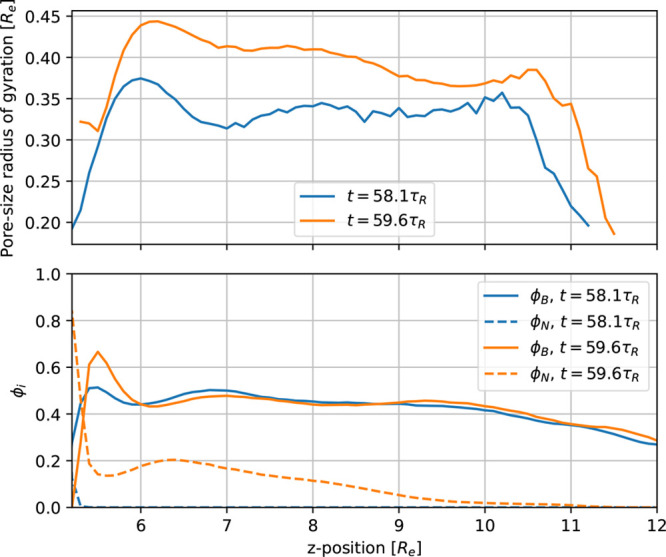
Top: Average lateral cylinder size at position, *z*, at the start of the NIPS process, *t* = 58.1τ_*R*_, and at *t* = 59.6τ_*R*_. Bottom: 1D density profiles of ϕ_*B*_ and ϕ_*N*_ at the corresponding times. The self-assembled top layer ends at *z* ≈ 11*R*_e_ for *t* = 58.1τ_*R*_ and at *z* ≈ 11.5*R*_e_ for *t* = 59.6τ_*R*_.

After performing EISA for 58.1τ_*R*_, we observe that a top layer of perpendicular cylinders with a thickness of approximately 6*R*_e_ has formed, and we commence the NIPS process. To this end, we represent the contact of the film with a coagulation bath by instantaneously exchanging the gas, *G*, with the nonsolvent, *N*. The nonsolvent is fully compatible with both solvents, *S* and *C*. As the solvent leaves the film, we convert it into nonsolvent when it reaches the conversion zone, at a distance *d*_NIPS_ = 1.3*R*_e_ above the film surface. The time evolution of the NIPS process is illustrated in Figure 6. We divided it into three stages:

(1) *Contact with the coagulation bath*: After exchanging gas to nonsolvent, there is a rapid decrease of the solvent densities at the film surface because the attraction between both solvents, *S* and *C*, and the nonsolvent is larger than the corresponding interaction with the gas. Moreover, the decrease of the distance between the film surface and the evaporation zone facilitates rapid exchange. Initially, the reduction of the solvent densities is compensated partly by a small retraction of the film surface (approximately 0.1*R*_e_ in a time of less than 1τ_*R*_) as the highly incompatible nonsolvent pushes the polymer skin slightly downward and partly by an increase of the local polymer density. This, in turn, gives rise to a reduced polymer mobility. The average mobility, *m*_*B*_, of the matrix block is presented in Figure 5b, calculated at the position of the maximal 1D density of ϕ_*B*_(*z*,*t*) . This shows that shortly after the contact with the coagulation bath, part of the polymer skin is arrested. This glassy arrest prevents further retraction of the film surface.

(2) *Transport of nonsolvent through the self-assembled polymer skin*: As solvent molecules leave the polymer film and are converted into nonsolvent, the film surface becomes immobile, and nonsolvent enters the polymer film. Since the nonsolvent, *N*, is less incompatible with the minority block, *A*, than with the matrix-forming block, *B*, *N* enters through the perpendicular cylinders. This gives rise to a slight swelling of the cylinder diameter. To characterize the diameter increase, we identify 2D *A*-rich clusters with threshold ϕ_*B*_ < 0.3 in a cross section at a given *z*-position and calculate their average radius of gyration. The results of this analysis are illustrated in Figure 7. Additionally, the pore size at the ultimate top of the film (in the first approx. 0.5*R*_e_) is slightly reduced compared to the pore size deeper inside the film. This conical pore shape already arises during EISA, *t* ≤ 51.8τ_*R*_. There, the preference of the gas molecules for the matrix-forming block, χ_*AG*_*N* > χ_*BG*_*N*, leads to an enrichment of *B* at the narrow film surface and a concomitant reduction of cylinder size in the ultimate vicinity of the film surface. Due to the chain connectivity, this decrease at the narrow film surface is compensated by a slight increase of cylinder diameter at a distance of order *R*_e_ further inside the film. At the start of the NIPS process, the rapid vitrification of the polymer at the film surface freezes the conical cylinder shape, independent from the preference of the nonsolvent. When the nonsolvent reaches the end of the self-assembled polymer skin, the final stage of NIPS commences.

(3) *Formation of the macroporous structure*: The interface between the perpendicular-cylinder structure and the disordered interior of the film is rather narrow. When the nonsolvent leaves the cylinders, small, nonsolvent-rich domains form at the interface between the self-assembled top layer and disordered interior of the film. Initially, these *N*-rich domains grow isotropically. Neighboring domains laterally fuse, resulting in a high lateral connectivity of macrovoids. The growing macrovoids deplete their surrounding of solvent and push away the polymer; i.e., the solvent densities near the interface between the nonsolvent-rich domains and the bulk of the film is reduced, and in turn, the polymer density is increased. The increased polymer density results in the self-assembly of the copolymer and, eventually, its glassy arrest. The latter effect limits the lateral growth of the nonsolvent-rich domains and, at later times, the macrovoids predominately elongate perpendicular to the film surface. For the parameters of the reference system, we can appreciate in Figure 6 that the front of the macrophase separation between the nonsolvent and polymer and the front of the microphase separation basically coincide.

**Figure 8:**
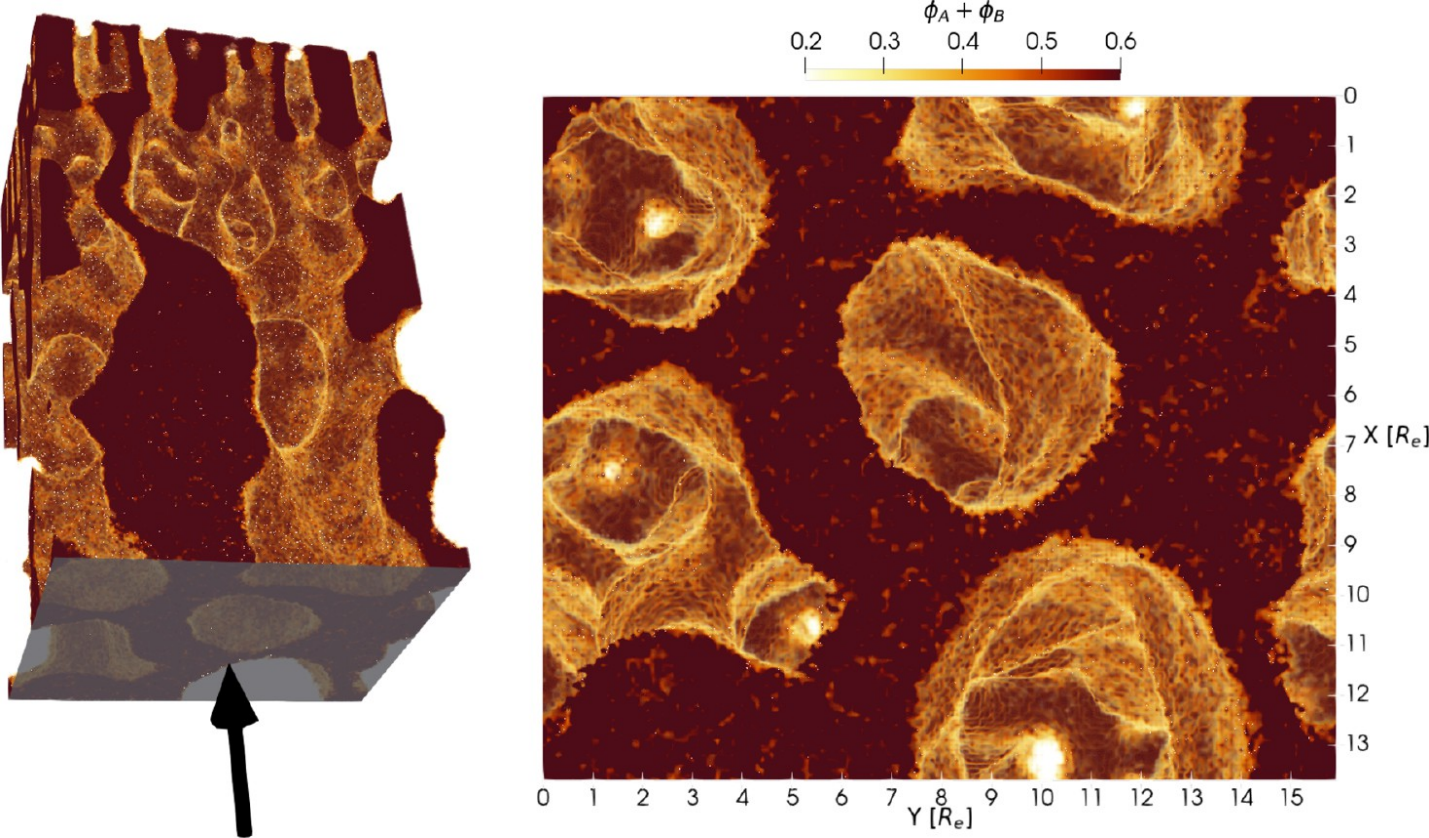
3D view of the total polymer density, ϕ_*A*_ + ϕ_*B*_, in the upper part of the membrane after NIPS for the reference system at time *t* = 114.8τ_*R*_. The left image shows a 3D snapshot of the structure, where the arrow and the plane at the bottom indicate the perspective from which the snapshot on the right was taken.

Finally, we comment on the continuity of the macroporous structure, which is necessary for an adequate flux through the membrane. To illustrate the continuity of the structure and its low tortuosity, Figure 8 shows the upper part, 0 ≤ *z* ≤ 32*R*_e_ of the macroporous substructure of the reference system. The snapshot, depicting the total polymer density ϕ_*A*_ + ϕ_*P*_, is taken at time *t* = 114.8τ_*R*_. The left image shows a 3D snapshot of the structure, where the arrow and the plane at the bottom indicate the perspective, from which the snapshot on the right is taken. This view from below shows that rather straight pathways from the bottom of the structure through the pores in the functional layer exist, illustrating the continuity of the structure and the existence of nontortuous pathways.

#### 3.1.3 Domain-Size Characterization

**Figure 9:**
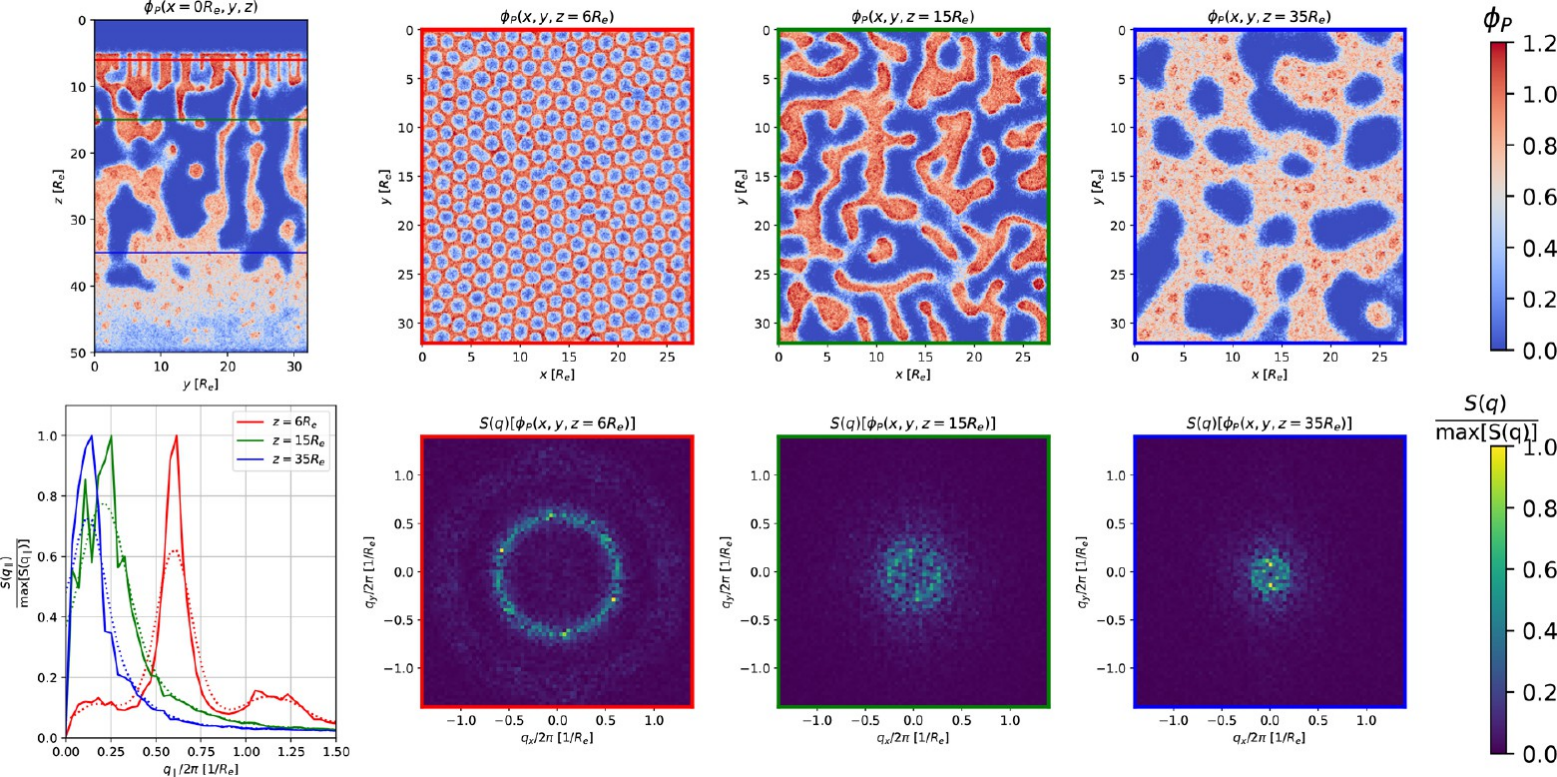
Lateral domain size of the macroporous structure after 58.1τ_*R*_ EISA followed by 58.1τ_*R*_ NIPS: The top-left image shows a cross section of the polymer density, ϕ_*P*_ = ϕ_*A*_ + ϕ_*B*_ in the *yz* plane. The three horizontal lines indicate the *z* positions of the polymer-density *xy* cross sections depicted in the three right panels. Bottom row: The leftmost image shows the radially projected collective structure factor, *S*(*q*_∥_,*z*), calculated in *xy* cross sections at the positions *z*/*R* = 6, 15, and 35. The dotted lines depict the corresponding smoothed *S*_*G*_(*q*_∥_) . The images next to it present the collective, 2D structure factor in the corresponding planes. The parameters are identical to those of the reference system, but the *xy* cross section is quadrupled, i.e., *L*_*x*_ × *L*_*y*_ × *L*_*z*_ = 27.6 × 32 × 50*R*_e_^3^.

An important characteristic of integral-asymmetric membranes is the gradient of the domain-size distribution; i.e., the self-assembled top layer provides selectivity, whereas the macroporous substructure provides mechanical support and retains a high permeability. To quantify the structure in *xy* planes at a fixed position, *z*, perpendicular to the film surface, we calculate the static, collective structure factor, *S*(*q*_∥_,*z*) in a system of quadrupled lateral area
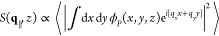
8This collective 2D structure factor, *S*(**q**_∥_,*z*), as well as its radial projection, *S*(*q*_∥_,*z*), are presented in Figure 9 for different layer positions, *z*. For *z* = 6*R*_e_, i.e., inside the microphase-separated top layer, the nonsolvent *S* is located at the center of the *A* cylinders. The 2D structure factor exhibits a ring, whose radius characterizes the dominant in-plane length scale. Additionally, one can appreciate 6 peaks on that ring, which indicate the long-range hexagonal order of the *A* cylinders. Deeper inside the film, *z* = 15*R*_e_ or 35*R*_e_, this long-range order of the polymer-density variations is lost, and *S*(**q**_∥_,*z*) is rather rotationally invariant in the *xy* plane. Nevertheless, the radially averaged *S*(*q*_∥_,*z*) exhibits a maximum, whose position quantifies the characteristic in-plane length scale, *d*(*z*)
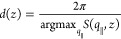
9Additionally, we calculate the smoothed structure factor, *S*_*G*_(*q*_∥_,*z*), by convoluting *S*(*q*_∥_,*z*) with a Gaussian of width σ_*q*_∥__ = 2π/*L*_*x*_. From its maximum we obtain the characteristic scale, *d*_*G*_(*z*).

**Figure 10:**
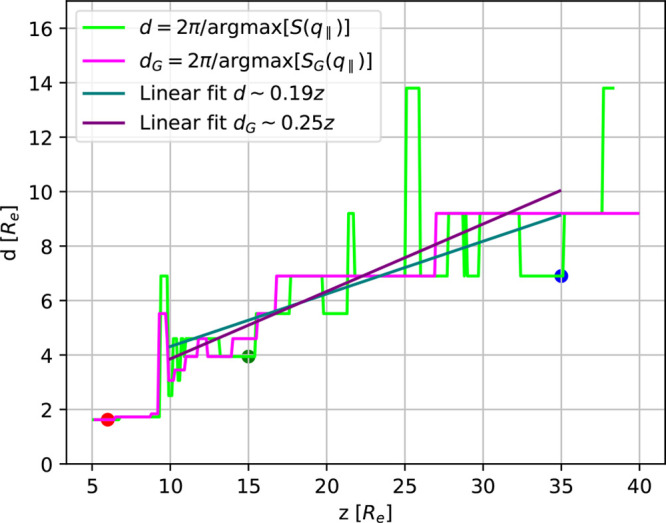
Domain size, *d*(*z*), according to eq 9, as a function of depth, *z*, as well as *d*_*G*_, obtained from the smoothed structure factor. Linear fits in the region 10*R*_e_ ≤ *z* ≤35*R*_e_ are indicated by lines. The three dots indicate the *z*-position, at which the cross sections are shown in Figure 9.

In Figure 10 we present the resulting *d*(*z*) and *d*_*G*_(*z*) with linear fits for the region from *z* = 10*R*_e_ to *z* = 35*R*_e_. These yield an approximation for the domain-size increase as a function of depth, Δ*d*/Δ*z* ≈ 0.19 and Δ*d*_*G*_/Δ*z* ≈ 0.25. Such an increase of the domain size has also been observed in recent field-theoretic studies.^26,33^ The effect, however, appears to be more pronounced for the parameters used in our particle-based simulations.

### 3.2 Parameter Variations

#### 3.2.1 Incompatibility between the Majority Component and Nonsolvent

**Figure 11:**
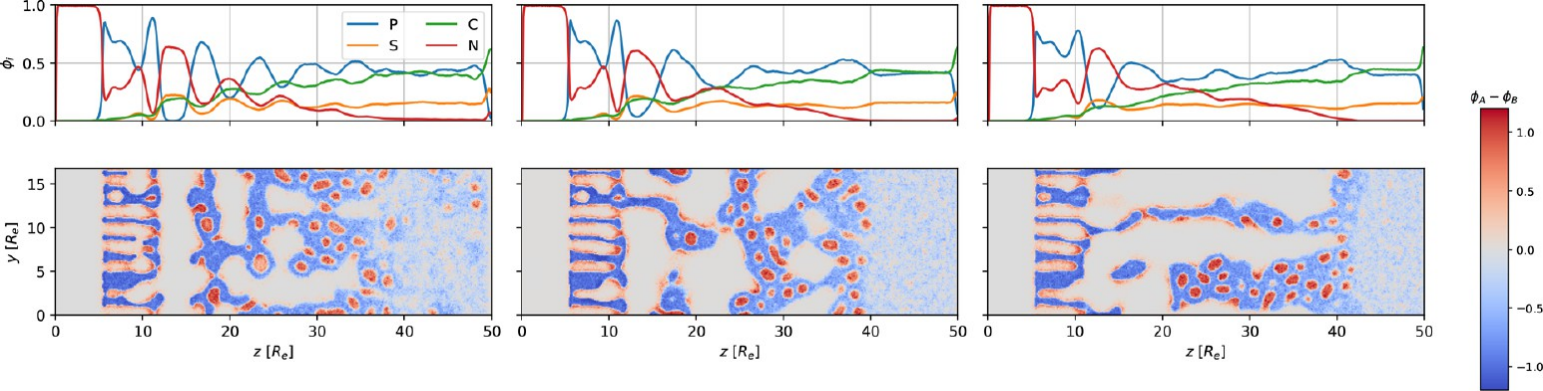
Top row: 1D density profiles of polymer, *P* = *A* + *B*, solvents, *S* and *C*, and nonsolvent, *N*, as a function of the perpendicular position, *z*, after 58.1τ_*R*_ NIPS. From left to right, the interaction between nonsolvent, *N*, and matrix block, *B*, increases, χ_*BN*_*N* = 100, 200, and 500, where the latter, rightmost system corresponds to the reference system. Bottom row: 2D cross section of the difference, ϕ_*A*_ – ϕ_*B*_, between minority-block and majority-block density corresponding to the 1D profiles above.

In Figure 11, we present density snapshots after 58.1τ_*R*_ NIPS for systems with different interactions between the nonsolvent and majority block, χ_*BN*_*N* = 100, 200, and 500. The *BN* incompatibility affects three regions:

(1) In the self-assembled top layer, we observe pronounced distortions of the cylindrical structure for small χ_*BN*_*N*. The small incompatibility between the nonsolvent and majority block allows some nonsolvent to enter the *B*-rich matrix, resulting in a fusion of neighboring *A*-core cylinders, and eventually nonsolvent domains may form inside the self-assembled top layer.

(2) The lateral extent of macrovoids—especially directly beneath the self-assembled top layer—increases with decreasing χ_*BN*_*N*. For χ_*BN*_*N* = 100 and the finite lateral system size studied, we even observe that the nonsolvent domain beneath the self-assembled top layer completely fills the *xy* cross section, resulting in a poor connectivity of the self-assembled top layer and the film. This can be rationalized by the macrophase-separation mechanism: During the early stages of NIPS, macrovoids form by the isotropic growth of initially small, *N*-rich domains beneath the self-assembled top layer. These *N*-rich domains form sharp interfaces with the polymer solution in the lateral direction. As the macrovoids grow, the polymer density at the internal interfaces increases, whereas the solvents become depleted, resulting in the glassy arrest of the polymer and preventing further lateral coarsening of *N*-rich domains. Instead, macrovoids elongate perpendicularly to the film surface because the solvent density and thereby the polymer mobility deeper inside the film remain high. A larger χ_*BN*_*N* gives rise to sharper interfaces between the nonsolvent and polymer, leading to a steeper increase of the *B* density, ϕ_*B*_, and a stronger reduction of the mobility. Thus, lateral coarsening stops earlier, resulting in a smaller lateral extent of macrovoids. Moreover, a large incompatibility between *N* and *B* also promotes the perpendicular elongation of *N*-rich domains along the *z*-direction because ϕ_*B*_ decreases with the distance from the film surface.

(3) For small χ_*BN*_*N* = 100, after a certain duration of NIPS, the microphase-separation front has progressed deeper than the macrophase-separation front. A small *BN* incompatibility permits some amount of nonsolvent to diffuse into the polymer solution (i.e., beneath the microphase-separation front). The *N* density inside the polymer solution is insufficient to provoke macrophase separation because the binodal and spinodal densities of *N* increase with decreasing χ_*BN*_*N*. As the polymer solution beneath the macrophase-separation front approaches the spinodal stability limit of macrophase separation, however, significant density fluctuations build up. This can be appreciated in the laterally averaged 1D polymer density profile, ϕ_*P*_(*z*), for χ_*BN*_ = 100, where fluctuations remain in the disordered region, 40 < *z*/*R*_e_ < 49 after laterally averaging over a finite system size. Inside these transient, large-amplitude density fluctuations, the local polymer density increases sufficiently for micelles to form. We note that for all parameters investigated, the minority component *A* forms the core of the micelles, although χ_*BN*_*N* ≫ χ_*AN*_*N* = 10. This is in accord with experimental observations.^16^

#### 3.2.2 Incompatibility between the Minority Component and Nonsolvent

**Figure 12:**
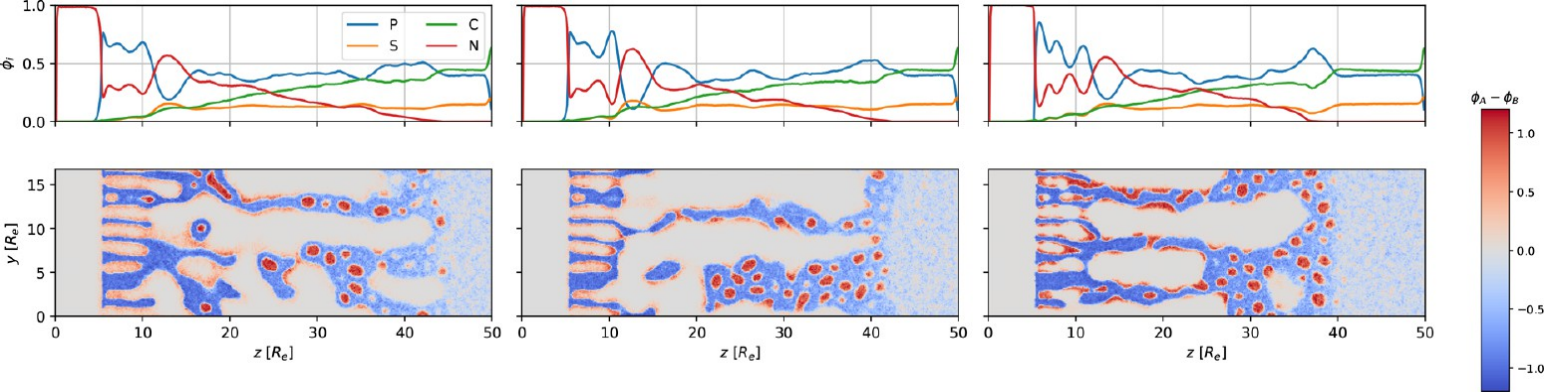
Top row: 1D density profiles of polymer, *P* = *A* + *B*, solvents *S* and *C*, and nonsolvent, *N*, as a function of the perpendicular position, *z*, after 58.1τ_*R*_ NIPS. From left to right, the interaction between the nonsolvent and the cylinder-forming block increases, χ_*AN*_*N* = 0, 10, and 25. χ_*AN*_*N* = 10 corresponds to the reference system. Bottom row: 2D cross section of the difference, ϕ_*A*_ – ϕ_*B*_, between the minority-block and majority-block density corresponding to the 1D profiles above.

Figure 12 presents density snapshots after 58.1τ_*R*_ NIPS for systems with different interactions between the nonsolvent and minority block: χ_*AN*_*N* = 0, 10, and 25. Again, we discuss the influence of χ_*AN*_*N* on different spatial regions:

(1) The self-assembled top layer is affected by the *AN* interaction in two ways: First, for large values of χ_*AN*_*N*, the selectivity of the nonsolvent for the *A*-core cylinder is small, and the nonsolvent already macrophase separates inside the self-assembled top layer, forming nonsolvent-rich domains that span multiple cylinders. This effect is similar to the observed distortion of the cylindrical domains for too small values of χ_*BN*_*N*, as described in section 3.2.1. Second, for small values of χ_*AN*_*N*, *N* is located at the center of the cylindrical domains, whereas *A* homogeneously coats the *BN* interface, shielding the unfavorable *BN* contacts. This homogeneous, lateral distribution for small χ_*AN*_*N* accelerates the NIPS process because the nonsolvent exchange through the *A*-core cylinders is faster. We observe that the nonsolvent front reaches the end of the self-assembled top layer for χ_*AN*_*N* = 0 in less than 1.5τ_*R*_ after the contact with the coagulation bath, whereas it takes more than 2τ_*R*_ for χ_*AN*_*N* = 25. Moreover, we note that, upon increasing χ_*AN*_*N*, the homogeneous coat of the *BN* interface laterally breaks up; i.e., the *A* blocks form the cores of micelles that are localized at the *BN* interface.

(2) Also, beneath the self-assembled top layer, for small χ_*AN*_*N*, a rather uniform coat of the minority block, *A*, forms at the interfaces between the nonsolvent and polymer in the course of macrophase separation. For the selected systems, the macrophase-separation front slightly lags behind the microphase-separation front; i.e., *A*-core micelles self-assemble in the film before macrophase-separation commences. As the polymer density inside the microphase-separated *A* cores is large, the mobility of copolymers participating in the self-assembly is reduced. For small χ_*AN*_*N*, this effect may delay the laterally homogeneous segregation of the *A* component to the energetically expensive nonsolvent–polymer interface. Indeed, closer to the film surface, where the macrovoids have formed earlier, we can appreciate an *A* coat of the *BN* interface, whereas deeper inside the film, the recently formed nonsolvent and polymer domains are less segregated, and the interfaces are not lined with *A* blocks. Large χ_*AN*_*N*, in turn, prevents a homogeneous *A* coat of the nonsolvent–polymer interface. Instead, in the course of macrophase separation, some *A* cores of micelles become localized at the nonsolvent–polymer interface due to the selectivity of the nonsolvent for the minority block. For large χ_*AN*_*N*, the interface between the nonsolvent and polymer is more strongly segregated than the *A*-coated interface at small χ_*AN*_*N*. Thus, the polymer mobility at the nonsolvent–polymer interface is smaller at large χ_*AN*_*N*. We hypothesize that this reduced mobility, in turn, prevents lateral growth of macrovoids. Indeed, a smaller lateral size of macrovoids at χ_*AN*_*N* appears to be compatible with Figure 12.

#### 3.2.3 Initial Polymer Density

We varied the initial polymer fraction, ϕ_*P*_0__, in the film by ±17% around the value of the reference system. Since ϕ_*P*_0__ affects the EISA, we have adjusted the processing parameters of EISA to obtain a well-ordered layer of perpendicular cylinders with a thickness of approximately 6*R*_e_, independent from ϕ_*P*_0__.

For the system with smaller ϕ_*P*_0__ = 0.344, the higher solvent density causes the system to cross the ODT later, and a longer EISA, *t* = 130.8τ_*R*_, than that for the reference system, *t* = 58.1τ_*R*_, is required to obtain a self-assembled top layer of approximately 6*R*_e_ thickness, although the evaporation flux of the volatile solvent is 9% faster than that for the reference system.

For the system with larger ϕ_*P*_0__ = 0.424, the ODT is rapidly crossed. For the reference value, χ_*SG*_*N* = 10, this gives rise to the lateral fusion of initially formed micelles into cylinders parallel to the film surface. Increasing the incompatibility between the volatile solvent and gas to χ_*SG*_*N* = 35 and increasing the distance between the film surface and the conversion zone to *d*_EISA_ = 2.5*R*_e_, we decrease the evaporation rate by about 40% and observed the formation of a 6*R*_e_-thick self-assembled top layer comprised of perpendicular cylinders after *t* = 87.2τ_*R*_ EISA.

**Figure 13:**
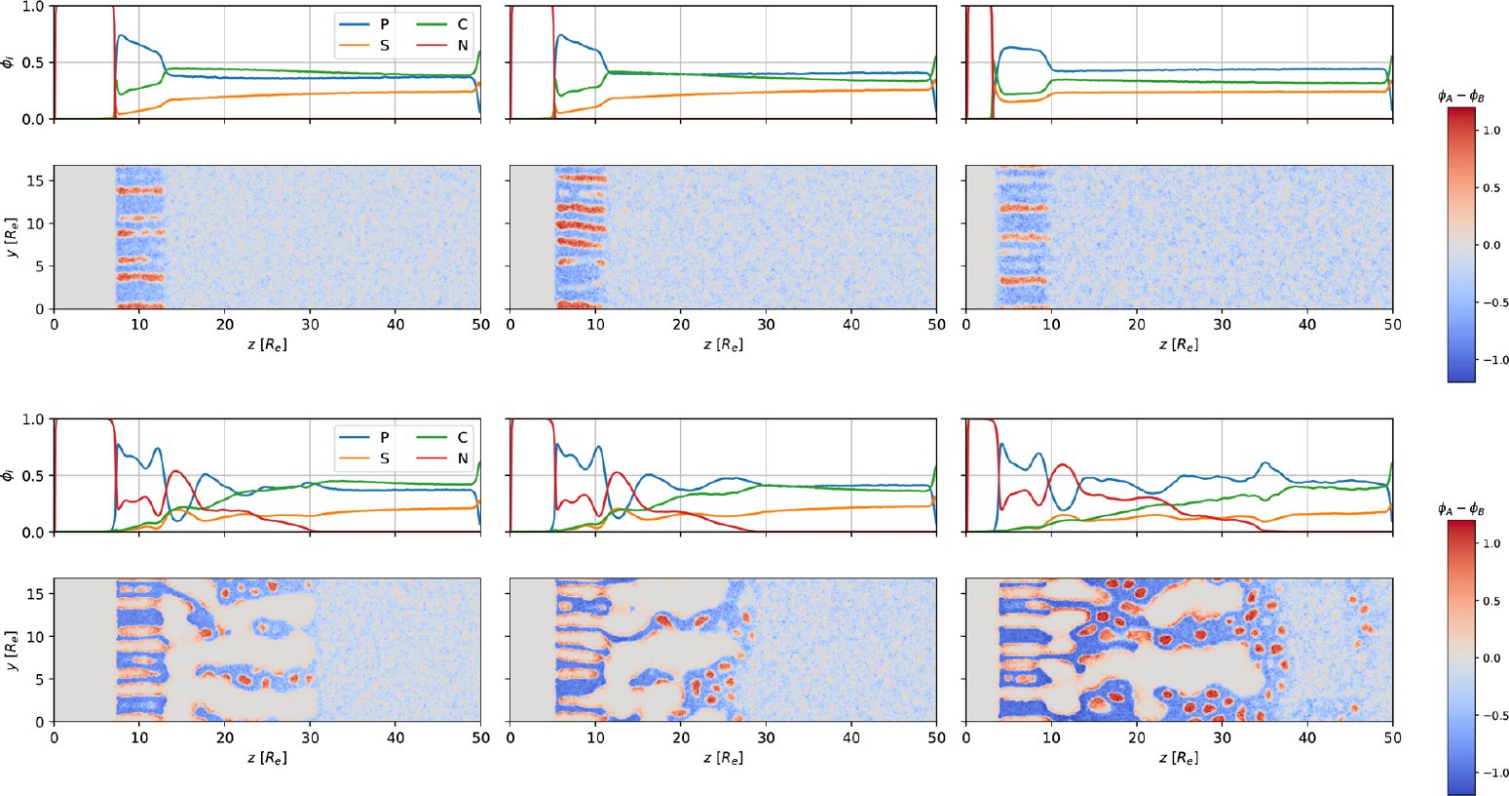
First and third row: 1D density profiles of polymer, *P* = *A* + *B*, solvents *S* and *C*, and nonsolvent *N*, as a function of the perpendicular position, *z*, at the end of EISA (first row) and after subsequent 23.3τ_*R*_ NIPS (third row). The initial amount of polymer, ϕ_*P*_0__, increases from left to right, ϕ_*P*_0__ = 0.344, 0.387, and 0.424, where the middle value corresponds to the reference system. Second and fourth row: 2D cross section of the difference, ϕ_*A*_ – ϕ_*B*_, between the majority-block and minority-block density corresponding to the 1D graphs above.

Laterally averaged profiles and snapshots for the different ϕ_*P*_0__ after EISA, which resulted in approximately 6*R*_e_-thick layer of perpendicular cylinders, are presented in the two upper rows of Figure 13.

1D density profiles and 2D snapshots for the different systems with varying ϕ_*P*_0__ after subsequent 23.3τ_*R*_ NIPS are presented in the bottom two rows of Figure 13. We choose a short duration of NIPS such that the rapidly progressing microphase-separation front in the system with the larger initial polymer density is not influenced by the finite system size, *L*_*z*_.

Again, we discuss the influence of ϕ_*P*_0__ in different regions:

(1) The self-assembled top layer remains intact in the course of NIPS for all chosen values of ϕ_*P*_0__.

(2) The lateral extent of the macrovoids directly beneath the self-assembled top layer decreases with increasing ϕ_*P*_0__. For the system with ϕ_*P*_0__ = 0.344 only a few, thin polymer connections are found between the self-assembled top layer and the macroporous structure. Similar to the explanation given in section 3.2.1, the lateral growth of initially small macrovoids directly beneath the self-assembled top layer is impeded by the reduced polymer mobility at the nonsolvent–polymer interface. An increased polymer concentration (due to a larger ϕ_*P*_0__) results in a faster decrease of polymer mobility in this region, resulting in a smaller lateral extent of macrovoids.

(3) ϕ_*P*_0__ controls the relative positions of the microphase-separation and macrophase-separation fronts. As we increase ϕ_*P*_0__, the ODT is reached earlier in the course of NIPS. Thus, in the case of large ϕ_*P*_0__ = 0.424, the microphase-separation front is approximately 3*R*_e_ in front of the nonsolvent–polymer-separation front after 23.3τ_*R*_ NIPS. While the position of both fronts approximately coincide for the reference system, we even observe the macrophase-separation front moving slightly ahead of the microphase-separation front for small ϕ_*P*_0__ = 0.344.

#### 3.2.4 Duration of EISA

Studying EISA *and* NIPS within the same simulation, we can investigate how EISA affects the subsequent NIPS process. In the following, we illustrate the role of time at which the self-assembling system is brought into contact with the coagulation bath.

**Figure 14:**
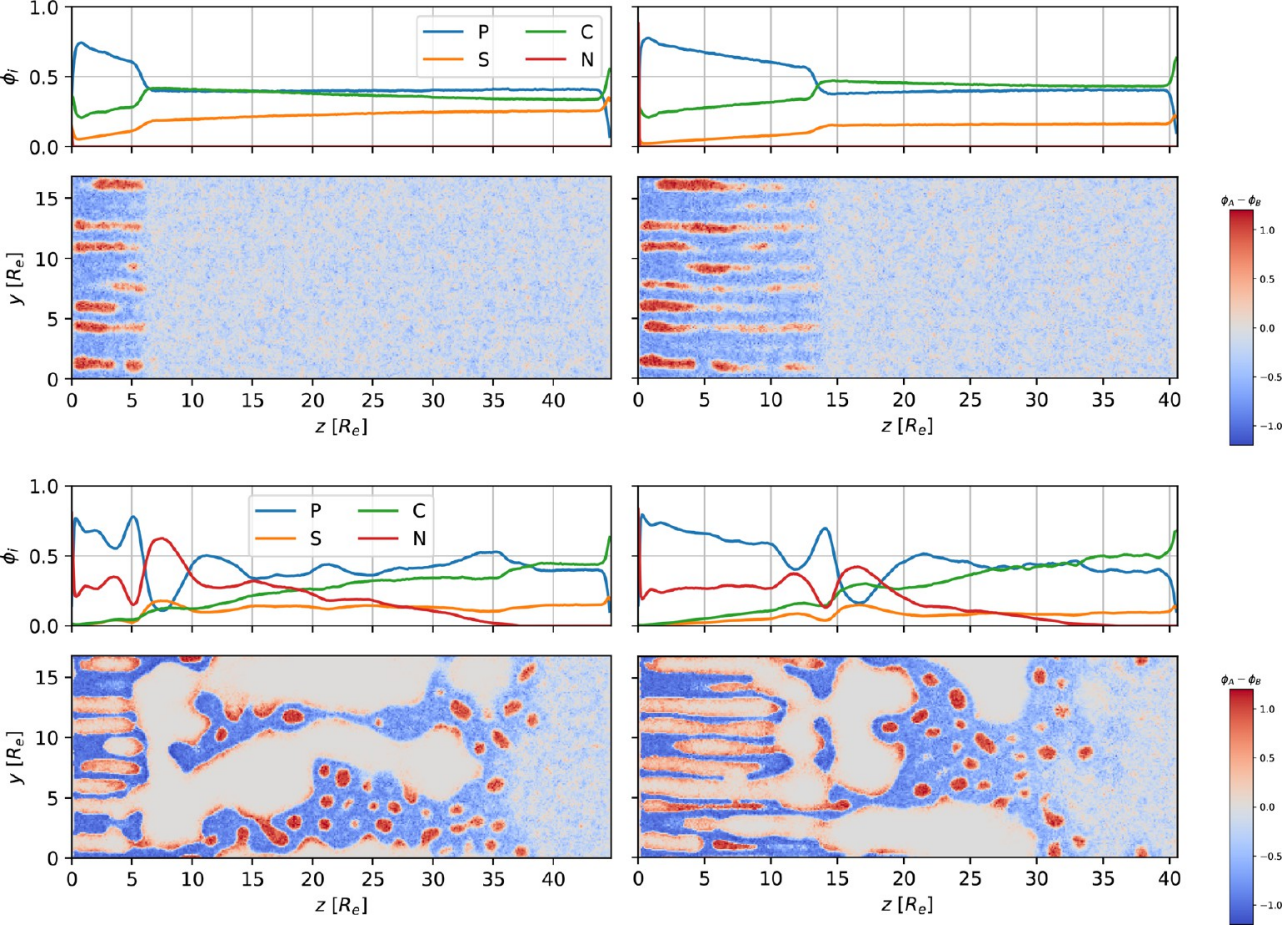
First and third row: 1D density profiles of polymer, *P* = *A* + *B*, solvents *S* and *C*, and nonsolvent, *N*, as a function of the perpendicular position, *z*, measured from the top of the film. The panels in the top row depict the beginning of the NIPS process after 58.1τ_*R*_ EISA (left) or 189τ_*R*_ EISA (right). The third row depicts profiles after 58.1τ_*R*_ NIPS. Second and fourth row: 2D cross section of the difference, ϕ_*A*_ – ϕ_*B*_, between the minority-block and majority-block density corresponding to the 1D graphs above.

In the first two rows of Figure 14, we present density snapshots for the reference system after 58.1τ_*R*_ EISA (left) and after 189τ_*R*_ EISA (right). The main differences due to a longer duration of EISA are the following:

The length of the self-assembled top layer is increased to approximately 13*R*_e_ for the longer EISA, compared to a length of approximately 6*R*_e_ for the shorter EISA (reference system). The cylinders at the end of the self-assembled top layer for the longer EISA, however, are not as segregated as those at the end of the self-assembled top layer after the shorter EISA. This is due to a smaller polymer concentration (and concomitantly larger concentration of solvents) at the end of the layer, after the longer EISA. Specifically, we find ϕ_*P*_ = 0.62 near the end of the short self-assembled top layer at *z* = 5*R*_e_ (measured from the film surface), whereas ϕ_*P*_ = 0.57 near the end, *z* = 12*R*_e_, of the long self-assembled top layer.

Additionally, in the disordered region beneath the self-assembled top layer, we observe for the longer EISA a smaller average concentration of volatile solvent, which is compensated by a larger concentration of nonvolatile solvent compared to that of the short EISA. The polymer concentrations do not significantly differ after the two EISA processes.

In the two bottom rows of Figure 14, density profiles and snapshots at time 58.1τ_*R*_ of the NIPS process, after the two different EISA durations are shown.

(1) The self-assembled top layer of the reference system with the shorter EISA stays mainly intact for its entire initial length of 6*R*_e_. In contrast, for the system with longer EISA, the self-assembled top layer is significantly distorted for *z* > 11*R*_e_; i.e., the initial thickness of well-ordered perpendicular cylinders has shrunk by approximately 2*R*_e_. In the course of NIPS, the nonsolvent enters the *A* core cylinders, and the concomitant distortion is not immediately impeded by the glassy arrest of the nonsolvent–polymer interface because of the larger solvent concentration at the cylinder ends after the longer EISA.

(2) Nevertheless, the lateral extent of macrovoids directly beneath the (remaining) self-assembled top layer remains small for the system with longer EISA because the polymer density in this region is still larger than ϕ_*P*_ beneath the self-assembled top layer in the system with short EISA. Analogous to the explanation given in section 3.2.1, the resulting decreased mobility leads to a smaller lateral extension of nonsolvent macrovoids beneath the self-assembled top layer.

(3) Additionally, we observe that the difference between the positions of the microphase-separation and macrophase-separation fronts is larger for the simulation with the longer EISA. The longer EISA results in a smaller concentration of the volatile solvent, *S*, and a concomitantly larger concentration of the nonvolatile solvent, *C*, at the beginning of NIPS. Since *C* is a worse solvent for the polymer than *S*, this promotes density fluctuations of the polymer and an earlier crossing of the ODT.

## 4 Summary and Outlook

We have used a single, particle-based simulation technique to investigate the sequence of both processes, EISA and NIPS. The simulations utilized a highly coarse-grained, particle-based model in conjunction with an efficient GPU-parallel implementation, SOMA,^35^ to study large length scales (up to 50*R*_e_ or m)) and long times scales (up to 200 *R*_e_^2^/*D* or s)) (using the estimates *R*_e_ ∼ 10^2^nm and *D* = *R*_e_^2^/τ_*R*_ ∼ 10^–8^cm^2^/s.) The sequential simulation of both processes in a single model with a fixed parameter set allows us to study the interplay of both processes in a setting that matches the experimental process of membrane fabrication via SNIPS.

We identify a reference set of structural, thermodynamic, and kinetic parameters that allows for a successful *in silico* fabrication of integral-asymmetric, isoporous diblock copolymer membranes. This parameter set in the high-dimensional parameter space of molecular architectures, interactions between different species, density-dependent mobilities, and processing parameters is neither adapted to a specific experiment nor tailored to optimize a particular membrane characteristic. We expect, however, that this reference system captures the salient, universal characteristics of SNIPS. We have independently varied selected parameters, leaving all other parameters unaltered. This highlights the role of a specific parameter on the final membrane morphology, demonstrates that our findings are robust with respect to small deviations from the reference set of parameters, and identifies a process window.

The simulations provide direct insights into the spatiotemporal structure formation and arrest. The role of the different thermodynamic (e.g., solvent selectivity for the block copolymer components) and kinetic (e.g., plasticizing effect of the solvent) characteristics is discussed.

The polymer solution initially contains *two* solvent species, one of which evaporates during the initial EISA process. In contrast to EISA with a single solvent,^22−25^ we observe that a preference of the volatile solvent for the minority block does not prevent perpendicular orientation of the self-assembled cylinder morphology. Moreover, the second solvent compensates the volatile-solvent gradient beneath the dense polymer skin, resulting in a sharper interface between the self-assembled top layer with well-ordered perpendicular cylinders and the disordered interior of the film. This reduces the thickness of the layer, where the polymer density is high enough to form micelles but insufficient to form cylinders, promoting the formation of perpendicular cylinders.^25^

In our reference system, the gas phase prefers the matrix-forming block *B*; thus, during EISA, the *A*-core cylinders are slightly narrower at the film surface than further inside the polymer skin. Although the nonsolvent that contacts the film at the start of the NIPS process prefers the cylinder-forming component, *A*, the rapid nonsolvent–solvent exchange and concomitant glassy arrest of the ultimate top of the film does not allow for a relaxation of the geometry of the cylinder tops; i.e., the cone-shaped opening of the cylinders is chiefly dictated by the interactions of block copolymer components with the gas, *G*.

The preference of the nonsolvent for the cylinder-forming species appears to be critical for the preservation of the self-assembled cylinder morphology in the course of NIPS. For large χ_*BN*_*N* or small χ_*AN*_*N*, the nonsolvent enters the film through the *A*-core cylinders. If the selectivity contrast of the nonsolvent is insufficient, however, the nonsolvent distributes more homogeneously in the self-assembled top layer and severely distorts the self-assembled structure by macrophase separation.

After the nonsolvent passed through the cylinders of the self-assembled top layer, macrophase separation between nonsolvent and polymer-rich domains commences, resulting in spongelike macrovoids. The lateral extent of these nonsolvent domains is controlled by the glassy arrest of the polymer-rich domains. An earlier arrest gives rise to smaller lateral domains sizes and is facilitated by narrower interfaces between the nonsolvent and polymer, i.e., large χ_*BN*_*N*, or larger initial polymer density.

For our reference system, the fronts of the microphase and macrophase separation basically coincide. If the incompatibility between the nonsolvent and polymer is smaller, however, some nonsolvent enters the polymer solution beneath the macrophase-separation front and induces fluctuations of the polymer density. Alternatively, polymer-density fluctuations may be enhanced by a larger initial polymer density, bringing the solution closer to the spinodal of nonsolvent–polymer macrophase separation. Under these conditions the polymer density may locally exceed the threshold for spherical micelles to form, such that the microphase-separation front progresses further inside the film than the macrophase-separation front. This effect, however, does not prevent the formation of integral-asymmetric block copolymer membranes.

We observe that the lateral size of macrovoids increases with distance from the film surface. For our reference system the characteristic lateral length scale increases by about a factor of 2 within a depth of 25*R*_e_. This is in qualitative accord with recent dynamic 2D SCFT calculations^26^ that report a somewhat weaker effect for comparable lateral size (approximately 32*R*_e_) but shorter times (approximately 12τ_*R*_).

Simultaneously studying EISA and NIPS by a single, particle-based simulation technique, we can investigate the interplay between the two processes. As an illustration, we studied the influence of the duration of EISA, observing that a later contact with the coagulation bath initially results in the formation of a thicker, self-assembled top layer but that this benefit is partially revoked in the course of NIPS.

Our study is a first step toward modeling the process-directed structure formation in these complex, multicomponent polymer materials. The interactions of our top-down, coarse-grained model are characterized by experimentally accessible parameters, such as *R*_e_ or the Flory–Huggins parameters. The particle-based simulations include thermal fluctuations and account for the interplay between the single-chain dynamics and the kinetics of the collective densities in a spatially inhomogeneous system. Thus, the simultaneous information about molecular structure, thermodynamics, and kinetics may provide guidance to experiments for controlling the complex interplay between system and process parameters.

To adapt our simulation model to a specific experimental system, the model parameters need to be identified—most importantly, the Flory–Huggins parameters and the density-dependent mobility coefficients. The Flory–Huggins parameters of an experimental mixture can be obtained, e.g., by measuring the interfacial tension between two demixed phases or by the scattering of composition fluctuations in a miscible system. One can estimate the density-dependent mobility of an experimental system by determining the diffusion coefficients of molecules in solutions at various compositions. For example, the polymer self-diffusion coefficient in a polymer–solvent mixture with varying solvent concentration yields information that is required to parametrize the polymer mobility as a function of ϕ_*S*_.

Although the use of a highly coarse-grained particle-based model and an efficient GPU-parallel simulation program, SOMA,^35^ enabled this study, the computational effort is significant, limiting a systematic optimization of SNIPS in the high-dimensional parameter space of SNIPS and studying NIPS for longer times and larger systems. Moreover, the present MC approach does not account for entanglements, hydrodynamic flow, or viscoelastic effects.^37^ These effects can be partially captured by slip-links^38,39^ and slip-springs^40,41^ or self-consistent Brownian dynamics simulations.^42−44^ The influence of these effects may be addressed in the future.
