# Correction to “Simulation of Membrane Fabrication via Solvent Evaporation and Nonsolvent-Induced Phase Separation”

**DOI:** 10.1021/acsami.4c02290

**Published:** 2024-02-21

**Authors:** Niklas Blagojevic, Marcus Müller

In the original article, the description of the mobility modifier in eq 6 is incomplete. The corrected description is
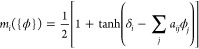
6with δ_*i*_ = 1, 1, 0, 0, 0, 0 for the different components *i* = *A*, *B*, *S*, *C*, *G*, *N*, respectively. This correction does not alter the conclusions.

